# Work, marriage and premature birth: the socio-medicalisation of pregnancy in state socialist East-Central Europe

**DOI:** 10.1017/mdh.2023.28

**Published:** 2023-10

**Authors:** Kateřina Lišková, Natalia Jarska, Annina Gagyiova, José Luis Aguilar López-Barajas, Šárka Caitlín Rábová

**Affiliations:** 1 Faculty of Social Studies, Masaryk University, Joštova 10, Brno, Czechia; 2 Institute of History, Czech Academy of Sciences, Prosecká 809/76, Praha 9, Czechia

**Keywords:** medical expertise, medicalisation, childbirth, reproductive health, gender, comparative history

## Abstract

Reproductive health in state socialism is usually viewed as an area in which the broader contexts of women’s lives were disregarded. Focusing on expert efforts to reduce premature births, we show that the social aspects of women’s lives received the most attention. In contrast to typical descriptions emphasising technological medicalisation and pharmaceuticalisation, we show that expertise in early socialism was concerned with socio-medical causes of prematurity, particularly work and marriage. The interest in physical work in the 1950s evolved towards a focus on psychological factors in the 1960s and on broader socio-economic conditions in the 1970s. Experts highlighted marital happiness as conducive to healthy birth and considered unwed women more prone to prematurity. By the 1980s, social factors had faded from interest in favour of a bio-medicalised view. Our findings are based on a rigorous comparative analysis of medical journals from Hungary, Poland, Czechoslovakia and East Germany.

## Introduction

State socialism needed employed women and expected them to also become mothers. Moreover, the women and their children had to be healthy. How was this to be achieved? Using the example of some of the most vulnerable—the babies born prematurely—and particularly the medical expertise built around them, we explore how women’s reproductive health was understood in Poland, Hungary, Czechoslovakia and East Germany over the course of state socialism. As mothers and children reproduce societies both physically and symbolically, analysing how healthy motherhood was conceived brings a nuanced picture of governance.

Prematurity is a particularly salient condition for several reasons. First, prematurity was a leading cause of infant mortality after the Second World War. East-Central Europe struggled with relatively high infant mortality. A downward turn in infant mortality rates was understood as an excellent indicator of a country’s overall improving health situation. Thus, prematurity has important quantitative and demographic effects. Second, prematurity constitutes a critical situation that might mean a life-or-death crossroads for the individual newborn and, if the child survives, could entail adverse consequences later in life. Therefore, prematurity has vital qualitative outcomes for each child and their family. Medical doctors wanted to understand the causes of premature births so that they could devise efficient prevention. Figure 1 shows prematurity rates in Czechoslovakia, East Germany, Hungary, and Poland, 1945-1989.^1^

While it is true that physicians point to somatic roots of medical conditions and prescribe pharmacological treatment and technological interventions, we identified two social causes that the medical experts, specifically gynaecologists and paediatricians, prominently linked to giving birth prematurely: women’s work and their marriages.^2^ We analyse what kinds of work experts connected with prematurity and what kinds of work were tacitly or explicitly excluded as causes of preterm birth. We discuss marriage, both in terms of the marital status of the mother, as her being unmarried was connected with an increased likelihood of giving premature birth, and in terms of marital satisfaction, since happiness in marriage was seen as paving the way towards healthy birth. Importantly, we argue that the perceived importance of work and marriage, as expressed by experts, shifted over time. These shifts, which we identified in the course of analysing expert discourses, do not follow the familiar periodisation of state socialism based on political changes, e.g. 1956 or 1968. However, we put the developments in expertise in a broader context. We show that in early socialism, experts agreed that paid employment did not threaten the pregnancy, in contrast to doing housework and being unmarried. In the 1960s, psychological stress came to the fore in expert analysis, in relation to both work and marriage. During the 1970s, work was rephrased as ‘socio-economic conditions’, and experts discussed work in connection with, for example, the mother’s education and housing. Social conditions were sidelined in the 1980s as biomedical approaches gained ground.

We understand medicalisation as a process in which conditions previously experienced or managed outside of medicine become part of the medical jurisdiction. Exemplified by a growing number and frequency of pregnancy check-ups and an increasing share of institutional births, typically in a hospital, we show how medicalisation was underway in our region from the beginning of state socialism. By the 1960s, medicalised pregnancy and birth had become the norm in East-Central Europe. For the first three decades of state socialism, we argue for socio-medicalisation: medical doctors paid close attention to the social aspects of women’s lives in an effort to prevent difficulties during pregnancy and birth. That interest faded in the 1980s, when new technologies and pharmacotherapies became available to reliably detect and treat pregnancy complications. We assert that it was no longer necessary for medical experts to identify complex social causes when they could turn to bio-technological solutions instead. As such, we label this new approach biomedicalisation. Both in definition and timing, it is in line with what Adele Clarke and her colleagues identified in the United States where

…around 1985, dramatic and especially technoscientific changes in the constitution, organization, and practices […] have coalesced into biomedicalization [which] emphasize[s] transformations of such medical phenomena and of bodies, largely through sooner-rather-than-later technoscientific interventions.’^3^

However, the biomedicalisation we analyse occurred outside of the market pressures that are part and parcel of the processes captured by Clarke *et al*.

Pregnancy care greatly expanded during the post-war period, although the levels of attendance and frequency of check-ups varied among countries. By the late 1950s in East Germany, Czechoslovakia and Hungary, over 80% of women were attending pregnancy care check-ups; in Poland, this percentage was only 60%, due to low pregnancy care scheme usage by rural women.^4^ The share of births occurring outside the home also grew from somewhat over a third in most countries at the beginning of state socialism (with Czechoslovakia a marked exception with already over 80% of births in a hospital) to over 95% in the 1960s (see Table 1).

**Table 1.** Share of institutional births^5^

	Poland	Hungary	East Germany	Czechoslovakia
Beginning of state socialism	35,6% in 1951	25,1% in 1946*	38% in 1950	81,5% in 1953
1960s	95,5% in 1968	96% in 1963	98% in 1965	96,2 in 1962

* Figures for Hungary in the early 1950s are not available.

In our paper, we first summarise historiographies of socialist medicine where we trace scholarly arguments about the character of medicalisation processes followed by the historiography of reproductive medicine in the four East-Central European countries under investigation. We continue by presenting our methodology and analysed sources. We then present our data divided into two sections: work and marriage. In each section, we follow the chronology and themes debated by the experts, offering an image of changing emphasis in medical expertise over time. In our paper, we focus on the category of gender, which we understand as operating on three levels, as theorised by West and Zimmermann:^6^ sex as a socially defined set of biological characteristics; sex categories as socially sanctioned identificatory displays; and gender as an activity, a ‘doing’ that occurs under the scrutiny of currently prevailing norms. Pregnancy and birth are particularly salient examples of the interplay between sex and gender levels. As such, we analyse what contemporaneous medical experts believed was an appropriate ‘doing’ for women to have a healthy pregnancy and birth. Moreover, we highlight how and when gender intersected with class and ethnicity.

## Historiography of medicalisation in East-Central Europe

In the broader field of the history of socialist medicine, scholars have highlighted an unprecedented surge in medicalisation after the Second World War. Some authors claimed the ‘medicalization of society’ meant that the state increasingly exercised social control, making the recruitment of medical professionals as state security agents more likely;^7^ or that the ongoing process of medicalisation and genetisation from the late 1960s allowed for increased ‘expert intervention into the private realms’ of people’s lives.^8^ Scholarship has argued that from the late 1960s and in late socialism, psychological factors, primarily stress, increasingly dominated debates on ‘health’ and ‘illness’.^9^

Others discussed persuasively that early socialist medicalisation either levelled the playing field for social actors or was strongly informed by social aspects of various medical conditions. Analysing the polio epidemic in 1950s Hungary, Dóra Vargha argued that the ‘medicalisation of patients’ lives’ triggered by the urgent health crisis opened possibilities for gender equality as well as the dissolution of class barriers and previous hierarchies in medicine.^10^ Researching Czechoslovak psychiatry, Sarah Marks showed that 1950s psychiatrists explained mental illness in connection with people’s social environment, never falling prey to mechanistic Pavlovian tenets.^11^ Polish scholars have explored the works of Magdalena Sokołowska, who developed an ‘environmental’ approach in her research on epidemics and conducted research on working women’s health in the broader context of the ‘double burden’, an approach she depicted as ‘socio-medical’.^12^ Scholars of East German medicine addressed how medical practices considered the social environment for tackling problematic life conditions and promoting proper habits. By the 1970s, though, state preventive practices had faded, and the responsibility for health was placed on the individual.^13^

Another subset of scholarship made a rather different argument: medicalisation of East-German social hygiene was swift,^14^ and social aspects came to the fore only later. According to Donna Harsch, ‘socialist health care was medicalized from its origins’ and became more ‘socially informed over time.’^15^ Related to sexuality, Dagmar Herzog argued that in the 1960s doctors paved the way for a more comprehensive healthcare system that accounted for the social in medicine.^16^ Similarly in Poland, as shown by Agnieszka Kościańska, sexology developed a holistic and interdisciplinary approach in the 1970s and 1980s that enabled viewing sexual disorders in a broader cultural and psychological context, in contrast to dominant biomedical and pharmacological trends in US sexology.^17^

## Historiography of reproductive medicine in East-Central Europe

There is a wealth of national case studies on reproductive medicine during state socialism, mostly focusing on abortion and contraception. This research has only very recently been placed in dialogue.^18^ Overall, existing studies argue that reproductive health was extensively medicalised, without paying close attention to how this process developed over time and without accounting for social factors in the medicalisation of pregnancy.

Many studies have been published recently about preventing or terminating pregnancies in Poland.^19^ Historians have discussed the state-socialist biopolitics that fuelled strong medicalisation since the early post-war period.^20^ This tendency of medical experts and the healthcare system was challenged only by alternative expertise, such as birth preparation classes, that nevertheless remained marginal.^21^ Agata Ignaciuk identified a shift in gynaecologists’ consideration of social and therapeutic indications for pregnancy termination in the 1970s.^22^

Scholarship on Czechoslovakia accentuated the increasingly technocratic nature of obstetric care, which offered few alternatives,^23^ and the eugenic aims of the socialist state^24^ that affected particularly Roma women who were excluded from general pronatalist policies.^25^ Some authors view granting abortion access to women as implicated in ‘a specific form of socialist governmentality [that] constituted the totalitarian communist state’ whose ultimate aims were a growing economic production and a growing population;^26^ others argued that experts recognised women’s right to decide about when to become a mother as fundamental for their equality.^27^

In East Germany, pregnancy and childbirth ranked among the most medicalised areas. Sabine Major highlighted the rapid development of state-run facilities, prenatal care and pregnancy counselling as parts of an ambitious reproductive health system.^28^ Donna Harsch discussed the transition from a state-centred system towards a more ‘liberal’ one in the 1970s, partially propelled by pressure from women.^29^ Eva Schäffler and others noted the state’s efforts to support the pregnancies of single and working women.^30^

In Hungary, as shown by Andrea Pető for the abortion trials of 1952–53, an informal network of medical professionals conducting illegal abortions undermined pronatalist state efforts, concluding that ‘female solidarity indeed triumphed over state coercion.’^31^ Comparing Hungary and Austria, Eszter Varsa emphasised eugenic similarities in promoting ‘quality’ and ‘healthy’ births throughout the Cold War period.^32^ Focusing on racialised aspects of medical discourses, Barna Szamosi viewed a continuity between pre-war and socialist times, as experts noted much higher rates of preterm births among the Roma minority.^33^ However, as Anna Borgos has shown, in the early 1960s Roma started to be identified in terms of their participation in the socialist workforce rather than their ethnic background.^34^

## Methods and sources

Methodologically, we use the sociology of expertise^35^ and time-sequencing approaches^36^ to analyse our expert-produced data. Following Gil Eyal, we view expertise as a ‘network linking together agents, devices, concepts, and institutional and spatial arrangements.’^37^ Within such networks, new understandings emerge, and with them new ways of defining people, managing problems and determining what kinds of people have what kinds of problems. This conceptual approach helps us see how causes of premature birth were viewed by experts or, in other words, what kinds of people were prone to give birth prematurely. Like ‘kinds of people’, problems are not given but evolve over time. To capture and make sense of their shifting nature, we follow time sequencing approaches that use reiterated problem solving, i.e. comparing historical times with altering modes of dealing with the ‘same’ issue.^38^ Since each epoch defines problems in a (slightly) different manner and mobilises various actors in order to arrive at a solution, one needs to ‘deploy contrasting cases to develop explanations both for period-specific solutions and for differences between periods.’^39^ These cases then connect into a narrative highlighting ‘historical switch points that are followed by more or less durable social regimes.’^40^ Jeffrey Haydu proposed organising narratives in time sequences and then showing how ‘characteristics or events at time 1 lead to or are transformed into characteristics or events at time 2.’^41^ This methodological approach was further developed in Andrew Abbott’s narrative program of research.^42^ Thus, we focus on trajectories and the ways in which they form and re-form over time.

We analyse scholarly writings of medical doctors, mostly gynaecologists and paediatricians, in four countries over four decades. We draw on key medical journals in each country, as well as scholarly books on prematurity and perinatal medicine published between 1946 and 1989. While sociologists, psychologists, and demographers were also involved, they contributed to these debates only marginally. Importantly, we noticed a significant level of interconnectedness in medical expertise: physicians stayed up-to-date with Western literature during the entirety of state socialism, including the 1950s; over time, they also increasingly cited their East-Central European socialist colleagues.

For East Germany, we identified *Zentralblatt für Gynäkologie* (Central Gazette for Gynaecology) and *Kinderärztliche Praxis und Zeitschrift für Ärztliche Fortbildung* (Paediatric Practice and Journal of Continuing Medical Education) as leading in their fields. For Poland, we analysed articles from *Pediatria Polska* (Polish Paediatrics) and *Ginekologia Polska* (Polish Gynaecology), as well as selected important articles from other medical journals. In Czechoslovakia, research into the causes of preterm births was addressed mainly by *Československá gynekologie* (Czechoslovak Gynaecology). Although the journal *Pediatrické listy* (Paediatric Letters), renamed *Československá pediatrie* (Czechoslovak Paediatrics) in 1955, concerned primarily the proper care and future development of premature babies, premature birth causes were also a topic. The issue of prematurity appeared in other publications, such as *Praktický lékař* (General Practitioner). In Hungary, gynaecologists and paediatricians published their research in the journals like *Magyar Nőorvosok Lapja* (Hungarian Journal of Gynaecology) and *Gyermekgyógyászat* (Paediatrics).

## Work

Particularly in the early post-war period, medical experts understood that purely somatic causes accounted for only some premature births; in a large percentage of cases, the causes were unknown. They searched for other factors that might be responsible, and their attention was drawn to work. The early socialist period was characterised by a rapid increase in women’s participation in paid employment and a heightened pace of industrialisation. Medical doctors surprisingly found that work was not a problem; in fact, they saw paid work as beneficial for women. This marked a sharp contrast with some western countries, where work was framed as harmful. Based on ‘extensive surveys’, a West German gynaecologist affirmed: ‘it is a well-recognized phrase in maternity care that pregnancy and work outside home are irreconcilable opposites’.^43^ Socialist experts, however, saw *housework* as problematic. Inspired by the utopian understanding that women should be freed from most household chores as a necessary precondition to emancipation, young socialist states were eager to provide collective solutions, such as canteens, washing services, and institutionalised childcare.

In East Germany, experts cited labour protection laws from the 1950s that significantly reduced the work obligations of pregnant women. East German experts found it ‘difficult to establish correlations between two such complex concepts as “premature birth” and social situation.’ The latter included work, a suspected cause of the high rate of premature births. However, the first studies conducted by gynaecologists indicated that ‘under the social conditions prevailing today, occupational work represents a burden which, taken alone, cannot produce an increase in the frequency of premature births.’^44^ The social conditions being referred to were the newly established socialist system, which in 1950 passed the ‘law of protection of mothers and children and the rights of women,’ seen as the culmination of a long struggle for women’s rights. ‘For women in the production process, pregnancy leave and weekly leave at the request of the mother, followed by annual leave, are laid down in the law’, wrote Hildegard Marcusson, a medical doctor and one of the proponents of the law.^45^ Similarly, Gerhard Opitz, a gynaecologist from Rostock and the leading researcher of the causes of prematurity, argued that thanks to the new law ‘there is no need to expect a considerable burden, especially at the end of pregnancy’.^46^

In the same period, Polish experts similarly saw work as unrelated to preterm birth. Most experts stressed that the potential negative impact of industrial labour on prematurity (toxic substances, heavy work) was eliminated by protection legislation passed in 1948.^47^ According to this legislation, pregnant women were moved to ‘lighter jobs’ from the sixth month of pregnancy, could not work night shifts or additional hours from the fourth month and could take up to four weeks’ leave before delivery.^48^ A leading expert in prematurity, gynaecologist Halina Hofman, conducted a retrospective analysis and concluded that employment had no link to preterm delivery,^49^ a conclusion further confirmed by another study published in 1956.^50^

In the early 1950s, Hungarian gynaecologists called for the introduction of strong central regulations and strict monitoring of adherence to protect pregnant women who were increasingly in the workplace. The experts understood that giving birth to infants preterm not only harmed society but also created a cost factor for the state:

Apart from other considerations, it is also financially disadvantageous, because the mother of a premature baby requires extended maternity leave, and the care of the premature baby itself is extremely expensive and does not always produce results.^51^

The implicit conclusion was that investment into work regulations for pregnant women would financially benefit the state.

When Czechoslovak experts discussed paid employment in the late 1950s, they agreed that premature births were caused by a woman’s fatigue from work, but not by the work itself. Based on a survey of women working in the South Bohemian region, gynaecologists concluded that:

When comparing the percentage of women employed in agriculture who perform strenuous and exhausting work (…) with women in other occupations, where the work is often less physically demanding, the difficulty of work does not seem to be the only decisive factor. It seems to us to be more a question of increased fatigue among employed women.^52^

Early state-socialist expertise even highlighted the positive effects of work. Polish paediatrician and leading expert in prematurity Izabela Bielicka argued that:

Women’s participation in economic, social and political life contributes to increasing welfare and health, including decrease in preterm birth. Professional employment, by providing woman independence and a sense of self-esteem, ensures, besides health, moral satisfaction and psychological balance, so important for a harmonious functioning of the physiological system.^53^

Working women also had better access to health care.^54^ In Hungary, experts noted the positive impact of canteens on nutritional levels in working women. While almost every second housewife consumed a cold lunch, factory workers in particular enjoyed full hot lunches that helped maintain the iron levels needed for a healthy pregnancy.^55^ Work was deemed especially helpful for the Roma minority who suffered preterm outcomes twice as often as the rest of the population. Gynaecologists observed that regular employment in both men and women had a positive impact on the pregnancy outcome. Experts related it to better housing and especially to the better balanced nutrition of the mother-to-be.^56^ It was repeatedly found that the nutrition of pregnant Roma women was deprived of important proteins and was consumed in insufficient amounts.^57^

Some types of work remained in the expert focus as complicating pregnancies and causing preterm delivery. Agriculture, which did not lend itself as easily to labour regulations as work in factories, came up often in expert writings. While agriculture was more demanding than other jobs, Czechoslovak experts blamed the rural lifestyles that the women had experienced since childhood. Poor hygienic conditions and a lack of exercise and rest had a much greater impact on preterm births than the work itself. Living in the countryside was therefore much more difficult than living in the city and put women at greater risk of health problems, including prematurity. In addition, women working in agriculture did not have the same benefits as other working women, specifically paid leave (nine weeks before childbirth), and placing them on light-duty work was not easy or even possible in agriculture.^58^ East German experts contradicted some pre-war studies painting a rather rosy picture of rural women and pregnancy. Instead, ‘it should be obvious to everyone that the agricultural women have not only worked the longest, but often also the hardest physically every day.’^59^ In studies conducted during the 1950s, women working in agriculture exhibited elevated rates of prematurity, which prompted experts to label agriculture ‘heavy work’.^60^ Curiously, the experts in more rural countries did not consider agricultural work among the potential causes of prematurity. Hungarian paediatricians directed attention to heavy physical work in industry, not agriculture, as a major cause for a spike in preterm birth around 1953.^61^ Similarly, Poland did not single out farm work, possibly due to a higher incidence of prematurity in urban settings.

The work that drew experts’ ire was housework and the ‘double burden’ employed women suffered. Czechoslovak experts discussed the issue in 1950. They agreed that housework was often physically very demanding: taking care of older children, running the entire household and carrying coal for heating could lead to premature births.^62^ Housewives in particular could become so overwhelmed by their duties that they did not take proper care of their health, which adversely impacted pregnancy and delivery.^63^ In a study of 1383 preterm births conducted at the maternity clinic in Brno, experts found that 19.6% of the women worked in factories and 14.4% in agriculture; the highest number of premature births, 51.2%, was found among housewives.^64^ It seems that while Czechoslovak experts identified fatigue as the main cause of premature births for work in industry or agriculture, housework was explicitly associated with physical effort and therefore considered more dangerous.

Polish experts similarly noted housework as more important in the aetiology of preterm birth than formal employment. According to a study published in 1958, in 40% of preterm births the mothers worked in white-collar jobs, the same percentage were housewives, and only 20% of women had blue-collar occupations. Heavy housework was identified as the cause of preterm birth in 8.5% of cases; employed work (white- or blue-collar) was 4.8%, although authors noted the dangers of exhausting commutes.^65^ An extensive study conducted in Łódź — a large centre of women’s employment in the textile industry — revealed that physical injury leading to preterm birth was much more frequently caused by ‘doing laundry, cleaning, moving furniture, tiredness’ than work outside the home. The authors concluded that ‘overburdening of women with housework and childrearing, combined with professional work, has some impact on preterm birth.’^66^

Hungarian doctors repeated that the ‘second shift’ was to blame, not physical work as such. A gynaecological study from 1964 looking at the parents of 1137 premature infants in Borsod County noted ‘that there were remarkably few agricultural workers (1.6%). The proportion of industrial (14.9%) and intellectual (14.8%) workers is almost the same, while 63.2% of women are in the household (“not working”).’ The authors specifically mentioned heavy work throughout the pregnancy as a major factor, without specifying where this work was carried out.^67^ An even broader study based on 3000 births conducted by the same gynaecologists concluded that work itself did not cause danger as long as female workers and their managers respected the protection laws. ‘Only 27.4% of our premature parents worked. Of these, 59% were in manual jobs and 41% in white-collar jobs. 73% of premature women were housewives, so-called “non-working women”.’ However, it was the stressful and nerve-racking domestic housework, especially when children were already present, that endangered the pregnancy.^68^ Gynaecologists lamented ‘the rush, noise and hustle and bustle of modern life, which bombards women with thousands of exogenous psychological traumas from the moment they start work in the morning, especially in cities,’ noting the ubiquitous side effects of urbanisation women could not escape from.^69^

By contrast, East German doctors in the 1950s dismissed the double burden as a primary cause of prematurity. In several instances, they drew on historical studies showing how prematurity decreased during the First World War even though women ‘often had to perform unusually heavy work’. Only when husbands returned home in 1918 did the preterm birth rates go up again.^70^ A comprehensive 1956 study by Gerhard Opitz found out that women with the double burden had mostly full-term deliveries and did not fare significantly worse than housewives, which made the experts conclude that ‘double occupation (Doppelberuf) does not have the effect one would expect’.^71^ In the 1960s, the double burden was hardly thematised.

In the late 1950s, experts began focusing on stress as another factor causing preterm births. Czechoslovak medical doctors were first to pay attention to sedentary occupations connected with ‘exhausting mental work’ (*vyčerpávající duševní práce*). They believed that women whose work required increased attention or those who worked in offices needed to use a higher brain activity that contributed to premature birth: ‘We assume that working in these occupations causes excessive load on the brain that is already weakened by pregnancy, and that this overload combined with other stimuli can cause premature birth’.^72^ Office lifestyle was, as doctors noted, often connected with smoking, which also negatively affected pregnancy. In a 1960 article, an occupational physician stated:

It seems that we have not been critical enough of the workload of female clerical and sedentary workers. We assumed that working at an office desk was the least risky for pregnancy. In this respect, we must change our opinion. (…) Exhausting mental work, which is often accompanied by excessive smoking, only increases the possibility of premature birth.^73^

In the 1960s until about the mid-1970s, psychological stress was on the listed causes of prematurity. In an interesting twist, even agricultural work was connected with stress. Hungarian gynaecologists noted that rural women outside state employment were usually engaged on an allotment plot. However, experts concluded that agricultural work as such did not increase the likelihood of preterm births but rather the psychological stress of their husbands being far from home pursuing industrial work.^74^ A study conducted by Hungarian gynaecologists in 1965 identified women in occupations who subjectively perceived their work as strenuous as having a higher incidence of preterm birth. In contrast to the assessment of objectively harming factors of employment, experts concentrated for the first time on the subjective perception of working mothers; by doing so, they stressed psychological well-being as decisive for birth outcomes.^75^

In East Germany, stress was mentioned immediately after the war^76^ as endangering the pregnancies of women ‘who had received sudden news of the death of a close relative, who were in prison, who were in the midst of family discord and severe financial hardship.’^77^ In the 1960s, based on East German and West German studies, doctors concluded that ‘the longer a stressful occupation was pursued during pregnancy, the more underweight the children are.’ Stress was seen not as solely resulting from work but from the ‘interaction of work, pregnancy, motherhood and domestic responsibilities.’^78^ Still, some professions were labelled as stress-inducing. These were mainly ‘heavy work’ in a physical sense, as some factories presented employees with preterm rates considerably higher than the national average. In the worst cases of physical stress, experts prescribed ‘strict bed rest’ and ‘removal from the work process’, which could even be accompanied by ‘prophylactic hospitalisation’. They called for the further expansion of pregnancy care to reduce occupational stress, and thus lead to ‘impressive reductions in preterm birth rates’.^79^

Polish experts did not generally discuss stress in relation to work as a possible cause for premature delivery. An exception was ‘nervousness in white collar jobs’ mentioned by a renowned Polish paediatrician in 1950. However, his opinions differed from the dominant argument, since he had a much more negative perception of all kinds of professional employment.^80^ In 1979, excessive stress was linked to employment in high managerial positions.^81^ Throughout the state socialist period, Polish experts focused far more on the potential physical impact of work than on psychological factors.

In the late 1960s, experts began to change their tune about the effects of paid employment on the outcome of pregnancy. Demographic research for 1967 and 1968 in Hungary showed that the proportion of premature babies was highest in non-agricultural manual working families and lowest in white-collar families. Premature births were more common in younger age groups among manual workers and in older age groups among women in white-collar jobs. By far the highest rates (twice as high as for white-collar workers) were found in women pursuing unskilled manual work as day laborers, domestic workers and office assistants. The demographers linked this phenomenon ‘not only to the nature of the occupation but also to its lower socio-economic status.’ To be sure, white-collar working women were contributing to high preterm rates in a different way: the negative impact of multiple abortions on birth outcomes was stronger than, for example, in the group of manual agricultural workers.^82^ Independently from the type of profession, a paediatric study from 1969 provided evidence for the positive effect of maternity leave taken before the due date. The data showed a strong correlation between the length of maternity leave and the baby’s good birth weight: among those who took leave one week before the due date, 15.3% had a preterm birth, contrasting with 8.0% at two weeks, and only 4% at three or four weeks. As expectant mothers had the freedom to decide on the starting point of the six-week maternity leave, which automatically extended into the post-natal period, starting points differed starkly. While women may have appreciated the flexibility of the system, experts clearly struggled with its possible side effects.

However, the authors of the same study made clear that work that was not marked by physical or mental strain was not at odds with a positive birth outcome. On the contrary, women appreciated a financial surplus in the family budget as beneficial for nutrition, housing, household appliances and overall lifestyle, which in turn had a positive impact on the development of the foetus. By making this argument, the experts implicitly noted the undeniable benefits of further mechanisation of household work and the advantages of Hungarian ‘fridge socialism’.^83^

By the 1970s, ‘work’ was often discussed in all our countries within the context of broader ‘socio-economic conditions’. In expert findings, socio-economic benefits stemming from women’s paid employment outweighed potential negatives. But at first, worries rose. During the 1970s, concerns grew about Budapest being a hotspot for the highest preterm rates in the country. While the picture varied dramatically throughout the city, Hungarian researchers identified the agglomeration of the capital city as especially representative of the problem. As the outskirts absorbed the newly arriving industrial workers pouring in from the countryside, living conditions were cramped. Gynaecologists of the Women’s Clinic at Semmelweis University of Medicine noted that women located in those areas experienced high levels of stress due to their long commute into the city as well as physical strain at their industrial workplace. To make matters worse, infrastructure for pregnancy care was poorly implemented exactly in those areas where it was strongly needed for improving exceptionally high preterm rates.^84^ Thus, the growing outskirts of Budapest were another infamous example of how infrastructure did not keep up with the pace of industrialisation, here posing new challenges to public health efforts promoted by experts.

Expert interest in socio-economic conditions also increased in Poland, triggered by a conference on prematurity held in the Polish Society of Gynaecology in 1974, when experts turned their attention to ‘external conditions’.^85^ Gynaecologist Roman Czekanowski argued that preterm birth was ‘a central issue of perinatology’ and ‘it is not only a medical problem, but also a socio-economic one.’^86^ By the beginning of the 1980s, experts had conducted various large-scale studies that took several factors labelled as ‘socio-economic’ or ‘socio-demographic’ into account, including age, education, housing conditions, and work. While women’s work in paid employment was widely believed to negatively impact their pregnancies—an argument often repeated in general medical journals—experts could hardly find evidence to support this claim.^87^ A 1979 study of 12 800 cases revealed that women classified as performing ‘heavy paid work and heavy housework’ or only housework had a higher incidence of prematurity, while ‘standard work and housework’ did not pose any danger in this regard, showing an acceptance of paid employment and the normalisation of the double burden.^88^ A team of gynaecologists from Poznań concluded that the incidence of prematurity was almost the same among working and non-working (in paid employment) women: ‘the results contradict the general opinion that working women give birth prematurely more frequently than non-working ones.’ Moreover, they discovered a link between work satisfaction and the length of pregnancy and noted that women found work to be attractive and economically positive as long as they were not ‘too ambitious’ in their struggle for professional advancement. Access to health care and protective legislation mitigated possible negative impacts of working conditions.^89^ The organisation of protective departments in industry mitigated the negative effects of such factors as night shifts, toxic substances and prolonged standing, although transportation to work remained a challenge.^90^ Another study, comparing work in different types of industry, found that the incidence of prematurity was very similar, and that potential risks were eliminated by proper pregnancy care.^91^ When one study revealed a higher incidence of prematurity among women working physically, experts explained that women with lower levels of education and rural women allegedly did not respect ‘the hygiene of work’, a term used to label workplace safety regulations.^92^

In Czechoslovakia, gynaecologists similarly suspected manual workers might suffer from premature births. However, research conducted in the Košice region revealed that women working in an iron foundry had the same prematurity rates as clerical workers and housewives. Gynaecologists explained these findings as a result of proper gynaecological care provided to factory workers directly at their enterprise, which helped to detect pregnancies even in the first or second month and involved transferring these women to lighter forms of work.^93^ The same study also noted a much higher rate of premature birth among Roma women and suggested that ‘increasing health awareness (*zdravotnícka uvedomelosť*) of citizens of gypsy origin’ was needed.^94^ Analysing the risk factors for prematurity, another group of gynaecologists in the same region also noted significantly higher levels among Roma women. These women dominated in the category termed ‘bad social circumstances’, leading doctors to conclude: ‘We understand an increased prevalence of premature births among gypsy inhabitants as a result of their lower health level and maturity.’^95^ However, the doctors did not elaborate on how the situation should be improved.

In various studies from the 1970s, East German experts also addressed class differences by including the ‘educational, social, and personal status’ of women. A study based on 6780 women concluded that there were no differences between working women and housewives in preterm delivery: both had a similar rate, around 6%. Yet, the experts were quick to add that social and economic factors provided working women with ‘considerably more favourable conditions than the social and educational status of families whose mothers do not work’.^96^ A further argument underscoring the difference between working women and housewives lay in ‘the attitude towards the (future) child’. Women who felt little or no joy about their future children had twice as many premature births as those who were looking forward to having a baby. Housewives tended to figure more in the negative attitude during pregnancy. In contrast, ‘the higher social and educational status of working women is associated with less frequent rejection of the child’.^97^ East Germans contrasted their findings with those from Britain where experts found a prematurity rate in working women double that of housewives. ‘Considering the literature, we had expected a higher rate of premature births among working mothers compared to housewives.’ In this light, the same rate among East German women both in and out of paid jobs was considered a success that experts explained as a benefit of women’s emancipation: ‘It has long been typical that in families with an above-average standard of living, the overwhelming majority of women are employed, and that the high standard of living is largely made possible by female occupation.’^98^

Indeed, it was in connection with labour protection laws and how well workplaces adhered to them that work was, however rarely, addressed from the late 1970s until the end of state socialism. East German doctors admonished employers that ‘in order to prevent premature births, it must be ensured that pregnant women at work comply with East Germany’s legal requirements in the workplace.’ Whenever a higher incidence of prematurity occurred, it was blamed on non-compliance, not on the nature of work itself.^99^

At the beginning of the 1980s, Hungary faced a unique situation following the new legislation which led to a blooming second economy. As a result, the population enjoyed major improvements in goods and services but not without a toll on the participants in the second economy. Negative health effects became widely discussed during the 1980s, and sociologists noted the harmful effects of entrepreneurial work on pregnancy. Due to the nature of self-employment, expectant mothers in the second economy were more likely to overwork themselves and ignore protective pregnancy regulations.^100^

By the 1980s, biomedicalised discourse became dominant, displacing work in its social aspects as a cause for preterm birth. This shift occurred first in Czechoslovakia in the late 1970s when experts turned their attention to women’s somatic conditions and aimed to prolong pregnancy using various drugs. Prematurity was formulated as a medical problem that could be successfully solved by hospitalisation and new types of mostly pharmacological treatment.^101^

When Polish gynaecologist Michał Troszyński brought up socio-economic conditions in 1981 during the 2^nd^ Symposium on Perinatal Medicine in Socialist Countries, his was a lone voice, since his international colleagues presented papers on biomedical aspects and technologies.^102^ Experts turned to biology as a powerful explanation for problems, even those connected with work. In a 1985 study, Hungarian gynaecologists tellingly reproached women for failing to choose occupations in line with their ‘biological make-up’. A lifestyle study based on a narrow cohort of 195 women in Budapest prompted gynaecologists to identify this failure as one of the causes for high preterm rates.^103^ In 1988, an East German gynaecologist admitted that ‘despite the many new findings and efforts presented here, we have to state that there have been no discernible changes in the underweight rate in our country over the last 15 years.’^104^ East German experts concluded there was not much they could do in the socio-economic realm to reduce prematurity. They then changed tactics and began to ‘search for pregnancies endangered by the threat of premature birth’, focusing on risky somatic conditions, such as diseases in pregnant women, low maternal age, or multiple gestations.^105^ Doctors could thus target a clearly defined medical problem, using pharmacological treatment to ‘shift the date of birth to a higher gestational age and to choose the optimal delivery procedure’ and after delivery rely on ‘the growing success of neonatological intensive therapy in the optimal rearing of such premature infants’.^106^ In this context, work barely appeared and only as a sidenote.

Even in Poland, where experts maintained an interest in socio-economic factors well into the 1980s, the biomedicalised approach also found its footing. In his handbook on preventing preterm delivery, Troszyński extensively discussed programs of intensive prenatal care to help identify risky pregnancies and to monitor and pharmacologically treat them in order to prolong the pregnancy.^107^ Socio-economic factors remained important since they influenced the assessment of risk, and various versions of medical documentation used in Poland took into account ‘difficult life conditions (*warunki socjalno-bytowe*)’, harmful work conditions, ‘psychic overburden’, and being unwed. Socio-economic conditions were therefore integrated into the new biomedical approach.

Work loomed large in the expert imagination, especially in early socialism when paediatricians and gynaecologists associated it with positive effects on pregnancy. The huge expert interest in the impact of work on pregnancy stemmed from the fact that women in East Central Europe entered the workforce from the late 1940s onwards on an unprecedented scale, and the presence of working mothers was far greater than it had been before the war. The socialist project of women’s emancipation through their advancement in paid employment involved debates on how women would reconcile their productive and reproductive roles. In the 1970s, experts identified a broader set of socio-economic conditions, such as women’s education levels and housing quality, which, if low, could negatively impact pregnancy. By the 1980s, biomedical approaches eclipsed social emphasis on either work or socio-economic circumstances.

## Marriage

Gynaecologists and paediatricians identified marital status as another non-somatic risk factor for giving birth prematurely. Marriage underwent dramatic legal changes at the beginning of state socialism, equalising the status of the wife and husband and of children irrespective of (non)marital birth.^108^ Compared to work, where changes were pressing on the daily level, legal changes to marriage might have taken longer to take hold, which accounts for the relatively smaller amount of attention from medical doctors. However, doctors frequently discussed the effects of marriage.

In Poland, doctors mentioned ‘unwed mothers’ as a traditional risk group in pre-war capitalist society, linking negative birth outcomes also to low working-class milieus. A retrospective study by Hofman revealed that all women from the ‘domestic workers’ group in her sample of premature deliveries were unmarried.^109^ Władysław Szenajch cited an early 19^th^ century intervention of the Medical Council of the Kingdom of Poland which observed a high incidence of premature birth and stillbirth among unwed women, and argued it had to do with the ‘harsh’ treatment of pregnant domestic servants. While neither author was explicit about it, their texts imply that under socialism such concerns were no longer relevant.^110^

In East Germany, gynaecologists argued that unmarried women delivered their babies preterm more often than their married counterparts due to ‘financial reasons and questions of home care’. In a Leipzig clinic, every fourth premature baby was born to a single mother.^111^ In the 1950s, East Germany implemented a strong program of pregnancy payment for every gynaecological check-up as an incentive for women to seek prenatal care. By the end of the decade, gynaecologists became alarmed that this system was driving women to collect the payments and then seek to abort late in their pregnancy, even after the sixth month.^112^ Doctors understood these late-term abortion attempts as failures that turned into premature delivery and concluded: ‘These changes are particularly noticeable among unmarried women; it is probable that the somewhat unfortunate payment system led to a temporary increase in late abortions (*Spätabtreibung*).’^113^

As prematurity decreased throughout the 1960s in East Germany, the connection among abortion, single women and prematurity vanished. Experts still saw unmarried women as more prone to preterm delivery, but by the 1970s marital status was framed within other socio-economic conditions rather than singled out as a key factor. Single women had a higher rate of prematurity, but the reason was not their marital status but cumulative factors such as being single, working long shifts and having poor economic conditions.^114^ Diminishing the weight of marital status came in line with the official discourse on single mothers, ‘who have always had the greatest share in infant mortality’ and since the 1950s were ‘particularly protected’ by East German laws.^115^ In other countries, while previous abortion was mentioned among causes of preterm birth, especially when the first pregnancy ended in abortion, it was always independent of the woman’s marital status.^116^

As discussed in connection with work, experts noted the harmful effects of stress, in this instance resulting from the less-than-ideal situation of an unmarried woman. Czechoslovakia was a forerunner in this respect as gynaecologists were discussing single mothers and stress already in 1950. They agreed that these women suffered from mental trauma due to their difficult life situations. Gynaecologists noticed that the numbers of preterm births among unmarried mothers declined if they were hospitalised because it reduced the pressures that single women faced in their daily lives.^117^

Causal connections with stress were typically drawn during the 1960s. In the Polish city of Łódź, the incidence of prematurity among unmarried women in the early 1960s was twice as high as among married ones.^118^ In 1967, gynaecologists argued that ‘being unmarried and pregnant is a prolonged/chronic psychological trauma (*przewlekły uraz psychiczny*), which negatively impacts the foetus. This trauma causes a higher incidence of premature deliveries.’^119^ Another expert observed that among ‘unknown causes’ in the analysed sample, almost 50% were women who were unmarried or married less than six months. He explained that being unmarried was either a circumstance provoking negative psychological impact or leading to attempted abortions that then led to preterm birth.^120^ Hungarian experts, on the other hand, lambasted the marriages of very young people as they ‘do not provide the conditions for young pregnant women to spend their pregnancy in a calm and balanced environment and to raise their babies in a harmonious and healthy way’ and result in a higher risk of preterm birth.^121^ Over the next two decades the problem accelerated, prompting sociologists to draw attention to the fact that average age of first time married women was descending. In comparison to previous decades, late-socialist Hungarian society experienced a drop in the average age of first-weds by two to four years. In a rather alarmist tone, experts noted that a third of women got married before reaching the age of twenty while 11% got married before their 18^th^ birthday. As the decision to marry was in most cases motivated by pregnancy, an exceptionally high percentage of preterm births found their way into the marriages of under-age mothers. By then, experts had acknowledged that the risk of giving birth prematurely or to low-weight infants was notably elevated in the age cohort of 14- to 17-year-old mothers. Based on the medical risk evaluation, sociologists were especially concerned that by the end of the 1970s around one in three children was born to mothers of exactly that age range, which contributed to relatively high preterm rates.^122^

From the mid-1960s and throughout the 1970s and 1980s, marriage became discussed in terms of its quality and its impact on pregnancy outcomes. In Hungary, both gynaecologists and paediatricians identified marriage as a significant psychological factor in two independent studies. Based on a cohort of 1227 women, a leading expert in prematurity, paediatrician László Velkey, showed that happiness and satisfaction lead to fewer preterm births as did a woman’s emotional acceptance of her pregnancy. Thus, pregnancy out of wedlock or unwanted pregnancy (either in the beginning or throughout) was more likely to result in preterm birth. While a combination of marital happiness and satisfaction with the financial situation in marriage were the ultimate precondition for positive birth outcomes, happiness overruled satisfaction in importance when only one of the two was present.^123^ Another study led by gynaecologists reached similar conclusions when identifying that a marriage where both partners were unsatisfied was an important culprit in negative pregnancy outcomes. Focusing on the inner dynamics of the emotional relationship between the parents, physical and psychological trauma during pregnancy were listed among the causes, just ahead of high alcohol consumption, which might hint at domestic violence with its detrimental effects. Overall, the results showed that emotional strain between partners caused high stress levels in the expectant mother, increasing the risk of preterm birth.^124^

In Czechoslovakia, the first research examining the psychosocial aspects of premature births that included partner relationships was conducted in 1974. Experts from various disciplines such as gynaecology, sociology, psychology and psychiatry listed the most common factors found in women who gave birth prematurely: dysfunctional family relationships, conflicts with co-workers, inability to adapt to the partner, educational differences between spouses and related feelings of inferiority, social isolation and deprivation of the woman, threat of possible divorce, housing distress and financial concerns of families with multiple children.^125^ Experts concluded that although these psychosocial aspects were very difficult to measure and assess, they had a major impact on prematurity.

The association between premature births and marital status was discussed anew in 1980s Czechoslovakia. Research conducted at the gynaecological and obstetrical clinic in the Czech city of Plzeň identified women under the age of 18 and unmarried mothers as the two main groups with a high incidence of preterm births. If a married woman in the study group gave birth prematurely, it was explained by a dysfunctional marriage: ‘The remaining women who gave birth were married but the marriage did not fulfil its function.’^126^ Disharmonic marriage was also openly associated with young women because their ‘marriage was more form than function’.^127^ Although the expert noted that young and unmarried women often faced economic problems, she mainly stressed that for a large number of them low education correlated with premature births. Interestingly, another research study did not link higher levels of education to socio-economic status but to increased harmony in the relationship, which was crucial for hassle-free pregnancy and birth. Compared to better educated women, those less educated were less aware of healthy lifestyles, did not use healthcare services as much and, above all, suffered from more problematic relationships. Experts believed that these women had more conflicts with their husbands and with their parents or co-workers, which may have contributed to giving birth prematurely.^128^ Of equal importance was the relationship of the pregnant woman to her expected baby: if the pregnancy was unwanted, the risk of premature birth increased.^129^

Similarly in Poland, experts continued to deem married women better positioned to give timely and healthy birth. However, a 1983 Poznań study revealed power relations in marriage and the broader family (when the young couple lived with the older generation) as important factors, leading the experts to conclude that ‘pregnant women who do not find support in their partner and the family are more likely to give birth prematurely than married women,’ and that ‘women who give birth prematurely were more dominated by the husband or parents than women who delivered on time.’^130^ They even noted that if a woman considered herself a ‘head of the family’, it had a beneficial impact on the length of pregnancy. Women who gave birth prematurely more frequently complained about their husband’s behaviour. ‘Getting married does not always mean that the woman changes her negative opinion about the partner,’ authors explained. In the 1980s, experts no longer considered only marital status as an indicator of social and psychological stability; they also paid attention to the quality of the relationship and power relations, linking them to birth outcomes.

Early socialist expertise typically continued to highlight the pre-war connection between unmarried status and negative pregnancy outcomes. Prematurity proved to be more prevalent among single women who, lacking a husband’s income, suffered from economic difficulties, increasing their stress and driving some to unsuccessful abortion attempts. Abortion, even after it was legalised, was a cause of the preterm delivery of subsequent pregnancies. The latter part of state socialism, beginning in the mid-1960s, was characterised by expert interest in marriage quality, not just marital status. Study after study showed medical doctors that women’s satisfaction or even happiness in marriage were great predictors of timely and healthy deliveries.

## Conclusions

Our analysis of expert understandings of the causes of prematurity during the four decades of state socialism shows that from the 1950s to the 1970s experts adopted a socio-medical approach that led them to consider the impact of work and marriage. Despite important differences between the four countries and changes in the understanding of both work and marriage, the socio-medical perspective clearly dominated by the end of the 1970s. It was then replaced by a bio-medical approach, although in Poland and Hungary socio-economic factors were still discussed well into the 1980s. While other scholars have discussed the rapid process of the medicalisation of reproductive health under state socialism, our research reveals that this process had two phases. This distinction changes our understanding of how medical discourse on biological reproduction shaped gendered ideas of healthy motherhood. We also uncovered the ways in which gender in these expert discourses intersected with class and, to some extent, ethnicity.

The approach to analysing class in manifestly classless societies is not straightforward. Nevertheless, we have shown that class tacitly appeared in expert writings when medical doctors considered the effects that paid labour—and its various types—had on pregnancy. In the 1950s, paid labour was typically seen as unproblematic, and experts often invoked the then-new labour protection legislation, affecting mostly blue-collar jobs, as a panacea for what in the capitalist past had taken a heavy toll on pregnancies. Indeed, experts were quick to highlight the positive effects of work, from women’s self-esteem to money, as insuring greater comfort. Farm work was, curiously, not addressed in the more rural countries of Poland and Hungary. It was rather the industrialised East Germany where experts labelled agriculture as heavy work and suggested bad pregnancy outcomes. Czechoslovak doctors similarly underscored poor hygiene in the countryside.

Housework was a frequent target of 1950s expertise as heavy lifting and caring for existing children compounded women’s exhaustion. Study after the study in Poland, Hungary and Czechoslovakia found housework problematic and even concluded that birth outcomes were worse for housewives than for working women. The double burden was thus seen as better than working solely in the household. Only East Germany was an outlier as its experts did not pay attention to housework.

The operative word of the 1960s was stress. From the late 1950s, Czechoslovak experts wagged their fingers at office work or generally sedentary work for exhausting women’s mental capacities. Hungarians noticed stress on rural wives whose husbands commuted to factories. In many instances, experts collocated ‘subjective’ with the word ‘stress’, especially when reporting on job-related stress that women ‘subjectively felt’. Marriage was discussed in terms of stress in the 1960s: Polish experts linked psychological trauma to the situation of unmarried women and Hungarians noted the unstable marriages of overly young couples.

In the mid-1960s, expert interest in the *quality* of marriage began. While in the preceding decade, marriage was discussed in terms of the woman’s marital status, from the mid-1960s marital happiness and satisfaction were viewed as vital. According to Hungarian studies, marital happiness even outweighed economic benefits in birth outcomes. Polish research from the early 1980s warned against the detrimental effects of power imbalances within marriage and even praised families where women were in charge. Czechoslovak studies from the same decade were markedly bleaker in tone when they highlighted family dysfunction and breakdown as conducive to preterm birth.

By the late 1960s, the classed discourse shifted to socio-economic status. Experts admitted that some kinds of paid work might have adverse effects but insisted that the benefits brought by women’s improved economic situation or higher education tended to outweigh the risks. Medical studies in unison concluded that the levels of premature birth between women in paid employment and housewives were identical, and sometimes even more favourable to employed women, and thus working women were not suffering due to their involvement in paid jobs. By the 1980s, work disappeared from the expert view. Experts began to focus on the individual woman’s biology and somatic diseases and turned almost exclusively to pharmacological and technological solutions.

Within the socio-medicalised discourse on the causes of prematurity, medical experts produced ideas about gender that had emancipatory potential for women. Their clinical practice and research implied a positive perception of paid employment and drew attention to traditional women’s duties such as housework or agricultural work as harmful for pregnancy. In the context of preterm birth, experts considered women’s psychological well-being and satisfaction in connection to work or to partner relations. Medical experts in state socialist countries, while often suspicious about the negative impact of paid employment, concluded that it was not, in fact, harmful and noted its positive aspects even during late socialism, which is often depicted as a period of ‘backlash’ regarding gender equality. Our research thus brings a nuanced understanding of gender deployed especially during late socialism.

The socio-medical approach had some limitations. It led to a near-constant preoccupation with marital status (with the exception of East Germany), perpetuating the enduring traditional stigmatisation of unwed mothers. Scrutinising the connection between productive and reproductive labour could strengthen the perception of working women as primarily mothers. Bringing stress into the analysis, experts at times undermined educated women’s positions, contributing to the argument about ‘excessive’ emancipation of women under socialism.

Socio-medicalisation could bring other categories into the picture such as ethnicity or class. As we have found, medical experts tended to understand ethnicity more in terms of socio-economic conditions, underscoring Roma women’s poorer nutrition, housing and employment situation, yet also blaming their lower health awareness without providing any course of action for improvement. Class, while never explicitly named as such, was present in expert writings. Early socialist optimism about the advantages afforded by working status was reflected in medical expertise on pregnancy in all four countries. Curiously, even in the 1970s when experts routinely examined all types of work, including physically demanding jobs, for potential adverse effects on pregnancies, they did not find worse outcomes for employed women than for their homemaker counterparts.

Having various limitations and nuances in mind, we show that socio-medicalisation had emancipatory aspects. In this sense, we show that the medicalisation of reproductive health, usually interpreted in negative terms as contributing to increasing control over women’s bodies, had a positive dimension.

## Footnotes

1 In the period in question, no official preterm birth statistics existed. The graph presents data gathered by the authors from the sources. The numbers may reflect either the percentage of preterm births (which often also included stillbirths) or the percentage of low-weight births (only livebirths), since the definition of prematurity shifted in national and international medical expertise (in the 1960s, it became based on the criteria of birth weight under 2500 grams and/or less than 37 weeks of pregnancy) and experts paid more attention to low birth weight as a better indicator. We indicate which kind of data are presented for each period. POLAND: For 1946–1950: prematurity rates based on one clinic in Warsaw: Halina Hofman, ‘Częstość występowania i przyczyny porodów przedwczesnych oraz wczesna śmierć noworodków niedonoszonych’, *Pediatria Polska,* 27, 6 (1952): 669–80. For 1951 (prematurity): Janina Sikorska-Kochanowska, ‘O przyczynach porodów przedwczesnych’, *Pediatria Polska,* 31, 8 (1956), 893–900. For 1961–1970 (low-weight births): Zygmunt Tesarz and Janina Holzer, ‘Noworodki żywo urodzone z niskim ciężarem ciała w Polsce w latach 1961-1970’, *Przegląd Pediatryczny,* 2 (1973), 85–90. For 1971–1977: Michał Troszyński, *Porody przedwczesne: przyczyny, zapobieganie, postępowanie lecznicze : przeznaczone dla pracowników oddziałów położniczych oraz lekarzy poradni dla kobiet*, Zeszyty Metodyczno-Organizacyjne (Warszawa: PZWL, 1981). For 1980–1987: Rządowa Komisja Ludnościowa , *Sytuacja Demograficzna Polski: Raport*, *Sytuacja Demograficzna Polski : Raport* (Warszawa: RKL, 1988). EAST GERMANY: For 1945–1952: Gerhard Opitz, *Über die Ursachen der Frühgeburt* (Berlin: Volk und Gesundheit, 1956). For 1954–1961: J. Brey and J. Wienold , ‘Welchen Einfluss nimmt die Sozialgesetzgebung auf die Frühgeburtlichkeit?’, Zeitschrift für ärztliche Fortbildung, 2, (1962), 79–84 . For 1969–1975: S. Akkermann *et al*. ‘Zum Einfluss sozialer Faktoren auf die Beziehungen zwischen mütterlicher Berufstätigkeit und Frühgeborenenquote’, *Zentralblatt für Gynäkologie*, 98, (1976), 193–99; Ch. Zwahr *et al*. ‘Zusammenhänge zwischen Berufstätigkeit und der Geburt von “Kindern mit niedrigem Geburtsgewicht”’, *Zentralblatt für Gynäkologie*, 100, (1978), 885–92. For 1980: Marianne Fuchs, Gertraud Hesse and G. Thiele, ‘Zu einem ideologischen Problem bei der Senkung der Säuglingsgssterblichkeit’, *Zeitschrift für ärztliche Fortbildung,* 74, (1980), 440–4. For 1985: Ernst Ludwig Grauel, ‘Frühgeborene haben größere Überlebenschancen. Symposium zur Senkung der Säuglingssterblichkeit’, *Berliner Zeitung*, 21 September 1975. HUNGARY: For 1945–1949: János Kubinyi, György Méhes and Dénes Haraszthy, ‘Adatok a koraszülés kóroktanához’, *Magyar Nőorvosok Lapja,* 14, 1 (1951), 8–17. For 1950–1988: Miklós Mikolás and Jenő Sárkány, ‘Az abortuszkérdésről - Négy évtized statisztikai adatainak tükrében’, *Magyar Tudomány,* 97, 7 (1990), 827–45. CZECHOSLOVAKIA: For 1945–1949, data based on a clinic in Brno: Miroslav Mrázek , ‘Ošetřování předčasných novorozenců’, Československá gynaekologie: časopis Gynekologické a porodnické společnosti, 15, 1 (1950), 151–715411699. For 1951: Karel Poláček and Gustav Syrovátka, ‘Péče o nedonošené deěti v Československu, její možnosti a úkoly’, *Pediatrické listy: orgán Československé pediatrické společnosti (sekce Čs. lékařské společnosti J.E.Purkyně),* 6, 5 (1951), 294–300. For 1953–1954, data from the Liberec Region: Otto Bradáč , ‘Podíl nedonošených dětí na natalitě a na kojenecké úmrtnosti v Libereckém kraji v letech 1953 až 1956’, Československá pediatrie, 14, 6 (1959), 555–6413671647. For 1955-1962: Miroslav Vojta, ‘Aktuální problémy příčin předčasných porodů’, *Československá gynaekologie: časopis Gynekologické a porodnické společnosti,* 29/43, 4 (1964), 249–54. For 1964–1965: Olga Štolová and Helena Dufková , ‘Současný stav a další vývoj péče o nedonošené děti’, Československá pediatrie, 23, 3 (1968), 193–975675535. For 1973: Karol Poradovský *et al*., ‘Pripomienky k niektorým zásadám lekárského vedenia predčasných pôrodov’, *Československá gynaekologie: časopis Gynekologické a porodnické společnosti,* 39, 5 (1974), 346–49. For 1975: data from a clinic in Banská Bystrica: Vladimír Plintovič et al., ‘Trend výskytu predčasných pôrodov v materiáli KÚNZ za 15 rokov’, Československá gynaekologie: časopis Gynekologické a porodnické společnosti, 42, 4 (1977), 304–5862070. For 1981, data from the West Bohemian Region: Jakub Mleziva et al., ‘Zdravotnické příčiny předčasného porodu’, Československá gynaekologie: časopis Gynekologické a porodnické společnosti, 47, 1 (1982), 47 . For 1984, data from the West Bohemian Region: Věra Rokytová and František Macků, ‘Průběh těhotenství u předčasného porodu: výběr z referátů přednesených na sjezdu Čs. společnosti gynekologicko-porodnické, Košice, 20.-22.9.1984’, *Československá gynaekologie: časopis Gynekologické a porodnické společnosti,* 50, 8 (1985), 584–85.

2 Experts also addressed other non-somatic factors such as education, housing, income, and family relationships, though less prominently than work and marriage. The first three factors were addressed in the 1970s as part of a broader set of socio-economic conditions, as we analyse in this paper.

3

Adele E. Clarke et al., ‘Biomedicalization. A theoretical and substantive introduction’, in A.E. Clarke et al. (eds) Biomedicalization: Technoscience, Health, and Illness in the U.S. (Durham: Duke University Press, 2010), 1–44 , https://doi.org/10.1215/9780822391258-001. For a recent take on women’s medicine and medicalisation see Caroline Rusterholz , *Women’s Medicine: Sex, Family Planning and British Female Doctors in Transnational Perspective, 1920–70*, *Social Histories of Medicine* (Manchester: Manchester University Press, 2020).

4 Czechoslovakia: 1952: 77% of pregnant women attended counselling centers (Zdeněk Štembera*, Rodička včera, dnes a zítra* (Praha: Maxford 2016), 35.) In 1956–1962, counselling centers had 83.4% of all pregnant women on record by the fourth month of pregnancy. (Augustin Syrovátka, Vladimír Srb, and Miroslav Vojta, ‘Přehled vývoje perinatální úmrtnosti v letech 1956–1962’, *Československá gynaekologie: časopis Gynekologické a porodnické společnosti,* 29/43, 6–7 (1964): 434.); East Germany: Frequency of check-ups: 1949: 3 times, 1954: 4 times, 1964: 5 times, late 1970s onwards: every month. In 1956, 84.9% of all pregnant women were already registered at the pregnancy counselling centre, but only 7.4% came for the first time by the 16th week. In 1964, 99% were registered and 91.3% came by the 16th week. (Tosetti 1967). Hungary: 1953: 8% women attended one time, 81% three times, in 1954: 4% one time, 81% three times, 1955: 4% one time, 86% three times (László Bukovinszky, ‘A terhesgondozás és a magzati károsodások megelőzése’, *Népegészségügy,* 38, 10–11 (1957), 271–4.). Poland: Women under pregnancy care schemes: 1951: 29%, 1954: 60%, rural women 15% (Archiwum Akt Nowych hereafter AAN, Ministry of Health, 1/27), 1970: 70% (almost 100% in urban areas, less than 30% in the countryside) (AAN, Komitet Centralny Polskiej Zjednoczonej Partii Robotniczej, LI 321).

5 The share in some countries, such as Poland and Hungary, is comprised of births in hospitals and in birth centres. For example, in 1951 in Poland, 5,5% of women delivered in birth rooms and 30,1% in hospitals; in 1968, it was 16% and 79,5%, respectively (based on: Irena Krysztofowicz, *Zwalczanie umieralności niemowląt* (Warszawa: PZWL, 1971), p. 53). László Váczy, ‘A magyar szülészet-nőgyógyászat huszonöt éve’, *Magyar Nőorvosok Lapja,* 33, 6 (1970), 481–9. Figures for East Germany from Statistisches Jahrbuch der Deutschen Demokratischen Republik (Berlin, Staatsverlag der Deutschen Demokratischen Republik, 1966), 556. Figures for Czechoslovakia in Miroslav Vojta, Lubomír Jíra and Augustin Syrovátka, ‘Úspěchy zdravotní péče o matku a dítě v Československu’, *Demografie: revue pro výzkum populačního vývoje,* 5, 3 (1963), 247.

6 Candace West and Don H. Zimmerman, ‘Doing Gender’, *Gender & Society,* 1, 2 (1987), 125–51, https://doi.org/10.1177/0891243287001002002.

7

Viola Lászlófi , ‘Doctors into Agents: The Technologies of Medical Knowledge and Social Control in State Socialist Hungary.’ The Hungarian Historical Review: New Series of Acta Historica Academiae Scientiarum Hungaricae, 10, 2 (2021), 328–56.

8

Hana Hašková and Radka Dudová , ‘Selective Pronatalism in Childcare and Reproductive Health Policies in Czechoslovakia’, The History of the Family, 25, 4 (2020), 627–648 . https://doi.org/10.1080/1081602X.2020.1737561.

9

Jan Arend , ‘Ist Stress westlich?: Zum zeitgeschichtlichen Ort der Belastungssorge’, Geschichte und Gesellschaft, 45, 2 (2019): 245–74, https://doi.org/10.13109/gege.2019.45.2.245.

10

Dóra Vargha , Polio Across the Iron Curtain: Hungary’s Cold War with an Epidemic, (Cambridge: Cambridge University Press, 2018)33315356; Dóra Vargha , ‘Socialist Utopia in Practice: Everyday Life and Medical Authority in a Hungarian Polio Hospital’, Social History of Medicine, 31, 2 (2018), 373–912999185310.1093/shm/hkx064PMC6018929.

11 Sarah Marks, ‘Ecology, humanism and mental health in communist Czechoslovakia’, in Mat Savelli and Sarah Marks (eds), *Psychiatry in Communist Europe* (Basingstoke: Palgrave MacMillan, 2015), 134–52.

12

Włodzimierz Piątkowski , *From Medicine to Sociology: Health and Illness in Magdalena Sokołowska’s Research Conceptions*, Studies in Social Sciences, Philosophy and History of Ideas 27 (Berlin: Peter Lang, 2020); Barbara Klich-Kluczewska and Katarzyna Stańczak-Wiślicz, ‘Biographical experience and knowledge production: women sociologists and gender issues in communist Poland’, in Anna Artwińska and Agnieszka Mrozik (eds.) *Gender, Generations, and Communism in Central and Eastern Europe and Beyond* (New York: Routledge, 2020). We refer to Sokołowska’s research further in this article.

13

Carsten Timmermann , ‘Americans and Pavlovians: the Central Institute for Cardiovascular Research at the East German Academy of Sciences and its precursor institutions as a case study of biomedical research in a country of the Soviet bloc (c. 1950-1980)’, in Kelly Loughlin and Virginia Berridge (eds), Medicine, the Market and the Mass Media: Producing Health in the Twentieth Century (London: Routledge, 2004), 25–65 ; Jenny Linek, *Gesundheitsvorsorge in der DDR zwischen Propaganda und Praxis* (Stuttgart: Steiner, 2016), http://www.igm-bosch.de/content/language1/downloads/bh59.pdf.

14 Gabriele Moser, *‘Im Interesse der Volksgesundheit …’: Sozialhygiene und öffentliches Gesundheitswesen in der Weimarer Republik und der frühen SBZ/DDR. Ein Beitrag zur Sozialgeschichte des Deutschen Gesundheitswesens im 20. Jahrhundert* (Frankfurt a.M.: VAS, 2002).

15

Donna Harsch , ‘Medicalized Social Hygiene? Tuberculosis Policy in the German Democratic Republic’, Bulletin of the History of Medicine, 86, 3 (2012), 394–423 2324191110.1353/bhm.2012.0059.

16

Dagmar Herzog , Die Politisierung der Lust. Sexualität in der Deutschen Geschichte des 20. Jahrhunderts (Munich: Siedler, 2005). 233–5.

17

Agnieszka Kościańska , Gender, Pleasure, and Violence: The Construction of Expert Knowledge of Sexuality in Poland, New Anthropologies of Europe (Bloomington, Indiana: Indiana University Press, 2020).

18

Eszter Varsa and Dorottya Szikra , ‘“New Eugenics,” Gender and Sexuality: A Global Perspective on Reproductive Politics and Sex Education in Cold War Europe’, The History of the Family, 25, 4 (2020), 527–49.

19

Agata Ignaciuk , ‘No Man’s Land? Gendering Contraception in Family Planning Advice Literature in State-Socialist Poland (1950s–1980s)’, Social History of Medicine, 33, 4 (2019), 1327–493346941110.1093/shm/hkz007PMC7805787. https://doi.org/10.1093/shm/hkz007; Sylwia Kuźma-Markowska and Agata Ignaciuk , ‘Family Planning Advice in State-Socialist Poland, 1950s–80s: Local and Transnational Exchanges’, Medical History, 64, 2 (2020), 240–663228463610.1017/mdh.2020.5PMC7120264, https://doi.org/10.1017/mdh.2020.5; Agata Ignaciuk , ‘“Clueless about Contraception”: The Introduction and Circulation of the Contraceptive Pill in State-Socialist Poland (1960s–1970s)’, Medicina Nei Secoli, 26, 2 (2014), 509–3526054213; Sylwia Kuźma-Markowska, ‘An Unexpected Continuity: Voluntary and Compulsory Sterilization in the Rhetoric of the Pre- and Post-World War II Polish Birth Control Movement’, *East Central Europe,* 38, 1 (n.d.): 97–114.

20 Sylwia Kuźma-Markowska, “Walka z „babkami” o zdrowie kobiet: medykalizacja przerywania ciąży w Polsce w latach pięćdziesiątych i sześćdziesiątych XX Wieku,” *Polska 1944/45-1989. Studia i Materiały*, 15 (2018), 189, https://doi.org/10.12775/Polska.2017.10; Ewelina Szpak, *“Chory człowiek jest wtedy jak go coś boli”: społeczno-kulturowa historia zdrowia i choroby na wsi w Polsce Ludowej*. (Warszawa: Instytut Historii imTadeusza Manteuffla Polskiej Akademii Nauk, 2018), 229–32; Barbara Klich-Kluczewska and Piotr Perkowski, “Obiekty biopolityki? Zdrowie, reprodukcja i przemoc,” in Katarzyna Stańczak-Wiślicz, Piotr Perkowski, Małgorzata Fidelis, Barbara Klich-Kluczewska (eds.) *Kobiety w Polsce 1945-1989: Nowoczesność, równouprawnienie, komunizm* (Kraków: Towarzystwo Autorów i Wydawców Prac Naukowych Universitas, 2020).

21 Agnieszka Kościańska and Agata Ignaciuk, ‘Re-Gendering Childbirth: Catholicism and Medical Activism Regarding Birth Preparation in Communist and Early Democratic Poland (1950s– 1990s)’, *Journal of the History of Medicine and Allied Sciences*, n.d.

22

Agata Ignaciuk , ‘In Sickness and in Health: Expert Discussions on Abortion Indications, Risks, and Patient-Doctor Relationships in Postwar Poland’, Bulletin of the History of Medicine, 95, 1 (2021), 83–112 3396710510.1353/bhm.2021.0003, https://doi.org/10.1353/bhm.2021.0003.

23 Ema Hrešanová, ‘The Island of Alternatives: Power, Medical Science, and “Gentle Birthing” in Socialist Czechoslovakia’, *Journal of the History of Medicine and Allied Sciences,* 73, 1 (2018): 73–95, https://doi.org/10.1093/jhmas/jrx056; Ema Hrešanová and Paula A. Michaels, ‘Socialist Science Across Borders: Investigating Pain in Soviet and Czechoslovakian Maternity Care’, *Revue d’Etudes Comparatives Est-Ouest,* 1, 1 (2018), 45–69, https://doi.org/10.3917/receo1.491.0045.

24 Radka Dudová, *Interrupce v České republice: zápas o ženská těla - Bibliografie dějin Českých zemí*, Vydání první (Praha: Sociologický ústav Akademie věd České republiky, 2012); Andrea Prajerova, ‘Developing the New Socialist Czechoslovak State: Abortion, Eugenics, and the Politics of Choice between 1945 and 1962’, *Canadian Slavonic Papers,* 61, 2 (2019), 203–24, https://doi.org/10.1080/00085006.2019.1596516; Jakub Rákosník and Radka Šustrová, *Rodina v zájmu státu: populační růst a instituce v českých zemích: 1918-1989*, Vydání první, Knižnice dějin a současnosti, 60. svazek (Praha: Nakladatelství Lidové noviny, 2016). Jakub Rákosník and Radka Šustrová, ‘Toward a Population Revolution? The Threat of Extinction and Family Policy in Czechoslovakia 1930s–1950s’, *Journal of Family History,* 43, 2 (2018), 189, https://doi.org/10.1177/0363199018759650.

25

Hana Hašková and Radka Dudová , ‘Selective Pronatalism in Childcare and Reproductive Health Policies in Czechoslovakia’, The History of the Family, 25, 4 (2020), 637 .

26

Radka Dudová , ‘Regulation of Abortion as State-Socialist Governmentality: The Case of Czechoslovakia’, Politics and Gender, 8, 1 (2012), 140 .

27

Kateřina Lišková , Sexual Liberation, Socialist Style: Communist Czechoslovakia and the Science of Desire, 1945–1989 (Cambridge: Cambridge University Press, 2018), 99–119 .

28 Sabine Major, *Zur Geschichte der außerklinischen Geburtshilfe in der Deutschen Demokratischen Republik*, Humboldt University, PhD Dissertation (Berlin, 2003); Donna Harsch, ‘Gendering Health Politics. East and West German Healthcare Systems in Comparison, 1950–70’, in Karen Hagemann *et al*. (eds), *Gendering Post-1945 German History: Entanglements* (Oxford: Berghahn Books, 2019), 136–59.

29 Donna Harsch, ‘The Fight against Infant Mortality in Cold War Germany: East/West Convergence on Liberal Governmentality’, *The Journal of Modern History,* 93, 2 (2021), 401–32, https://doi.org/10.1086/713997.

30 Eva Schäffler, ‘Ostdeutsche Paarbeziehungen aus der Perspektive der Reproduktion: Schwangerschaftsabbruch und Kinderbetreuung’, in E. Schäffler (ed.) *Paarbeziehungen in Ostdeutschland*. *Auf dem Weg vom Real- zum Postsozialismus* (Wiesbaden: Harrassowitz Verlag, 2017), 219–78, https://doi.org/10.2307/j.ctv11sn50d.8; Anna Kaminsky, *Frauen in der DDR* (Berlin: Ch. Links Verlag, 2016); Edith Ockel, ‘Gesundheit der Frauen und Gesundheitspolitik in der DDR’, *Jahrbuch für Kritische Medizin*, 24 (1995), 105–21.

31

Andrea Pető , ‘Women’s Rights in Stalinist Hungary: The Abortion Trials of 1952-53’, Hungarian Studies, 29, 1–2 (2002), 49–7617233136.

32

Eszter Varsa , ‘Sex Advice East and West: Sex Education and Family Planning in Cold War Austria and Hungary’, The History of the Family, 25, 4 (2020): 649–70.

33 Barna Szamosi, ‘Public Health Concerns Regarding Reproduction Structured by Race/ Ethnicity and Class during State Socialism in Hungary’, *Pro&Contra,* 3, 1 (2019), 47–66.

34

Eszter Varsa, ’“The (Final) Solution of the Gypsy-Question:” Continuities in Discourses about Roma in Hungary, 1940s–1950s’, Nationalities Papers, 45, 1 (2017), 114–30.

35 Gil Eyal, ‘For a Sociology of Expertise: The Social Origins of the Autism Epidemic’, *American Journal of Sociology,* 118, 4 (2013), 863–907, https://doi.org/10.1086/668448.

36

Andrew Delano Abbott , Time Matters: On Theory and Method (Chicago: University of Chicago Press, 2001); Andrew Delano Abbott, *Processual Sociology* (Chicago: University of Chicago Press, 2016); Jeffrey Haydu, ‘Making Use of the Past: Time Periods as Cases to Compare and as Sequences of Problem Solving’, *American Journal of Sociology,* 104, 2 (1998), 339–71, https://doi.org/10.1086/210041.

37 Eyal, o*p. cit.* (note 35), 863.

38 Haydu, *ibid.*

39 Haydu, *ibid.*, 356.

40 Haydu, *ibid.*, 349.

41 Haydu, *ibid.*, 351.

42 Abbott, *op. cit.* (note 36).

43

Hans Naujoks , ‘Soziale Probleme der Schwangerschaft’, Soziale Welt, 1, 1 (1949), 79–88 .

44 Opitz, *op. cit.* (note 1), 20-21.

45

Hildegard Marcusson . Schutz von Mutter und Kind. Der gesunde Säugling, das Kind und seine Pflege (Berlin: Volk und Gesundheit, 1952), 17 .

46 Gerhard Opitz and H. Noack, ‘Das Frühgeburtenproblem beleuchtet an Hand von 57000 Geburten’, *Zentralblatt für Gynäkologie,* 26 (1954), 1009–22.

47 Rajmund Barański and Izabela Bielicka, ‘Opieka nad wcześniakami w Polsce jako problem społeczny’, *Pediatria Polska*, 4 (1948), 507–512; Izabela Bielicka, *Metodologiczne wytyczne organizacji opieki nad wcześniakami* (Warszawa: PZWL, 1953).

48 For a discussion on the relationship of women’s employment and reproductive health in public debates in post-war Poland see Małgorzata Fidelis, ‘Equality through Protection: The Politics of Women’s Employment in Postwar Poland, 1945–1956’, *Slavic Review,* 63, 2 (2004), 301–24, https://doi.org/10.2307/3185730.

49 Halina Hofman, ‘Czẹstość wystẹpowania i przyczyny porodów przedwczesnych oraz wczesna śmierć noworodków niedonoszonych’, *Pediatria Polska*, 27, 6 (1952): 669–80 (note 1).

50 Sikorska-Kochanowska, *op. cit.* (note 1).

51 János Kubinyi, György Méhes and Dénes Haraszthy, ‘Adatok a koraszülés kóroktanához’ *Magyar Nőorvosok Lapja,* 14, 1 (1951), 8–17, here: 16.

52 Jan Příbrský and František Drnek, ‘K otázkám příčin předčasných porodů v pohraničních jihočeských okresech’, *Československá gynaekologie: časopis Gynekologické a porodnické společnosti,* 23/37, 4 (1958), 278.

53

Izabela Bielicka , Zagadnienia z patofizjologii, leczenia i pielęgnowania wcześniaków w świetle spostrzeżeń własnych (Warszawa: PZWL, 1951), 32–33 .

54

Magdalena Sokołowska , ‘Metody pracy zespołu leczniczo-sanitarnego przy Zakładach Przemysłu Bawełnianego im. Stalina w Łodzi’, Zdrowie Publiczne, 6 (1954), 460–7414374687.

55

László Velkey , ‘A koraszülés veszélyei’, Természettudományi Közlöny, 97, 5 (1966), 215–21.

56

János Pázmányi , ‘A koraszülések társadalmi vonatkozásai.” Magyar Nőorvosok Lapja, 28, 5 (1965), 281–85.

57
*Ibid;* Péter Bősze and Antal Volosin , ‘A Karcagi magas koraszülött frequentia okairól’, Orvosi Hetilap, 108, 36 (1967), 1693–966070229.

58 K. Fellnerová, ‘Předčasné porody žen pracujících v zemědělství: předneseno na krajském semináři v Prostějově v únoru 1955’, *Československá gynaekologie: časopis Gynekologické a porodnické společnosti*, 21/35, 2 (1956), 131.

59

Karl-Heinz Mehlan , ‘Sozialhygienische Probleme der Frühgeburt’, Zeitschrift für ärztliche Fortbildung, 3 (1954), 89–97 13169971.

60 Opitz, *op. cit.* (note 1), 21.

61

Emil Stark , ‘A koraszülöttkérdés Járási kórházban’, Gyermekgyógyászat, 6, 8 (1955), 249–5613285741, here: 252.

62

Věra Jeřábková , ‘Přehled předčasných porodů na II. porodnické klinice KU v Praze’, Československá gynaekologie: časopis Gynekologické a porodnické společnosti, 15, 1 (1950), 46, 49 15411680.

63

Fr. Havránek and A. Horníček , ‘Přehled předčasných porodů na porod. odd. st. obl. nem. v Uh. Hradišti.’, Československá gynaekologie: časopis Gynekologické a porodnické společnosti, 15, 1 (1950), 63 15411682.

64

Bohuslav Holásek , ‘Přehled předčasných porodů na porodnické klinice M. U. v Brně’, Československá gynaekologie: časopis Gynekologické a porodnické společnosti, 15, 1 (1950), 52 15411681.

65

I. Brzozowska and Z. Brzdękiewicz , ‘Analiza przyczyn porodów przedwczesnych’, Ginekologia Polska, 29, 3 (1958), 245–5513598080.

66

Alina Komorowska and Maria Królikowska , ‘Wpływ pracy na przebieg porodów przedwczesnych i niewczesnych’, Medycyna Pracy, 3 (1961), 226 .

67

István Argay , Tivadar Nemecskay and Zsigmond Szombathy , ‘Borsod megye 1964. évi össz-koraszülésének elemzése, majd 4 év múlva a koraszülöttek után vizsgálata’, Magyar Nőorvosok Lapja, 33, 2 (1970), 186–91, here: 187 .

68 Velkey, *op. cit.* (note 55).

69 Pázmányi, *op. cit.* (note 56), 283.

70 Mehlan, *op. cit.* (note 59), ‘92.

71 A. Weimann, *Finden sich Unterschiede in der Reife der Kinder bei berufstätigen und nicht berufstätigen Schwangeren?* (Dresden: Medizinische Akademie, 1969).

72 Jiří Grégr, ‘Předčasné porody v textilním průmyslu’, *Československá gynaekologie: časopis Gynekologické a porodnické společnosti,* 22/36, 1–2 (1957), 136.

73 Rudolf Skokan, ‘Předčasné porody u zaměstnaných žen’, *Praktický lékař: časopis pro další vzdělávání lékařů,* 40, 23 (1960), 1091–92.

74 Pázmányi, *op. cit.* (note 56).

75

Jenő Dömötöri , Sándor Csömör , István Berbik , Attila Horváth and Mária Polgár , ‘Koraszülések demográfiai vonatkozásai klinikánk 5 éves anyagában’, Magyar Nőorvosok Lapja, 39, 3 (1976), 271–75, here: 273 .

76 In 1950, Czechoslovak experts conducted a retrospective study of premature births between 1945 and 1949. In 1945, they recorded a higher incidence of premature births in April and May, especially among women of German nationality, resulting from the uncertainties at the end of the war. Holásek, *op. cit.* (note 64), 53. In Hungary, experts have also directed their attention to stress: Pázmányi, *op. cit.* (note 56), 281–85.

77 Opitz, *op. cit.* (note 1), 15-16.

78 Akkermann et al., *op. cit.* (note 1), 195.

79

J. Rothe and H. Stiezel , ‘Zur Funktion der Schwangerenerholungsheime als integrierter Bestandteil der pränatalen-präpartalen Betreuung in der DDR’, Zeitschrift für die gesamte Hygiene, 16 (1970), 300–303 5522957.

80

Władysław Szenajch , ‘Wcześniactwo’, Pediatria Polska, 24, 5–6 (1950), 452–6914780739.

81

Rozalia Osuch-Jaczewska and Jan Tomala , ‘Wpływ pracy zawodowej kobiet na przebieg ciąży i porodu oraz na stan, dojrzałość, dystrofię i umieralność noworodków’, Ginekologia Polska, 50, 2 (1979), 135–45422061.

82

Egon Szabady , ‘A koraszülöttek arányának időbeli, nemzetközi és társadalmi rétegek szerinti összehasonlítása’, Demográfia, 14, 1–2 (1971), 45–52 .

83 László Velkey, “A terhes-, csecsemő- és gyermekvédelem társadalmi kérdései.” *Borsodi Szemle*, 13, 1 (1969), 45–54.

84

Jenő Dömötöri , Sándor Csömör , István Berbik , Attila Horváth and Mária Polgár , ‘Koraszülések demográfiai vonatkozásai klinikánk 5 éves anyagában’, Magyar Nőorvosok Lapja, 39, 3 (1976), 271–75. This was already cited in footnote 75.

85

Witold Michałkiewicz et al., ‘Problematyka badawcza porodu przedwczesnego’, Ginekologia Polska, 50, 12 (1979), 1057–58317058.

86

Roman Czekanowski , Poród przedwczesny (Warszawa: PZWL, 1980).

87

Andrzej Zdziennicki , ‘Praca zawodowa kobiet jako czynnik zagrożenia płodu’, Wiadomosci Lekarskie 41, 10 (1988), 652–543070942.

88 Rozalia Osuch-Jaczewska and Jan Tomala, ‘Wpływ pracy zawodowej kobiet na przebieg ciąży i porodu oraz na stan, dojrzałość, dystrofię i umieralność noworodków’, Ginekologia Polska, 50, 2 (1979), 135–145.

89

Jan Grzesiak , Tadeusz Pisarski and Andrzej Woźnica , ‘Społeczno-medyczne aspekty wcześniactwa. IV. Porody przedwczesne w aspekcie zawodowych problemów kobiet’, Ginekologia Polska, 54, 1 (1983), 45–86862255.

90

Andrzej Zdziennicki , ‘Problem wcześniactwa w oddziale pracy chronionej. I. Dojazdy do pracy’, Ginekologia Polska, 54, 8–9 (1983), 543–76667885.

91

Danuta Bromirska and Stanisław Różewicki , ‘Ocena porównawcza częstotliwości występowania porodów przedwczesnych u kobiet zatrudnionych w zakładach o różnym profilu pracy’, Medycyna Pracy, 37, 2 (1986), 101–63747829.

92 Michał Troszyński, ‘Wybrane czynniki demo-społeczne ryzyka w ciąży w porównaniu z masą urodzeniową i umieralnością okołoporodową płodów i noworodków’, in *II Symposium of Perinatal Medicine of the Socialist Countries: Poznań, November 19th-21st.*, 1981, 59–60.

93

M. Sedliak , Jaromír Holler , and J. Šteso , ‘Výskyt rizikových faktorov pri predčasných pôrodoch u tehotných pracujúcich v ťažkom priemysle a poľnohospodárstve’, Československá gynaekologie: časopis Gynekologické a porodnické společnosti 39, 5 (1974), 332 4844820.

94
*Ibid.,* 332.

95

Š. Turek , E. Tejbusová and J. Šimko , ‘Predčasné pôrody z hladiska rizikových faktorov’, Československá gynaekologie: časopis Gynekologické a porodnické společnosti, 39, 5 (1974), 330 4844819.

96 Akkermann *et al*., *op. cit.* (note 1), 195-197.

97
*Ibid.,* 195; S. Akkermann , Die Säuglingssterblichkeit nach Vollendung des 10. Lebenstages (Berlin: Volk und Gesundheit, 1970).

98 Zwahr *et al.*, *op. cit.* (note 1), 890.

99

Ch. Zwahr et al., ‘Mehrdimensionale Untersuchung zur Häufigkeit von “Kindern mit niedrigem Geburtsgewicht” unter Berücksichtigung von Alter, Parität und Berufstätigkeit’, Zentralblatt für Gynäkologie, 101 (1979), 1015–24532429; Zwahr et al., *Ibid.*, 885–892. ‘Zusammenhänge zwischen Berufstätigkeit und der Geburt von „Kindern mit niedrigem Geburtsgewicht”’.

100 Antal Bőhm, ‘A többszörösen hátrányos helyzetű rétegek vizsgálata’, *Szociológia*, 3–4 (1981), 279–332.

101 Jozef Babič and A. Zadjorova, ‘Náš postup pri liečbe hroziacích a začínajúcich sa predčasných pôrodov’, *Československá gynaekologie: časopis Gynekologické a porodnické společnosti,* 42, 4 (1977), 302–302; V. Kölblová, ‘Prevence předčasného porodu tokolýzou’, *Československá gynaekologie: časopis gynekologické a porodnické společnosti,* 42, 4 (1977), 302–3; Jozef Pakan *et al*., ‘Sledovanie účinku tokolytík u hroziacich predčasných pôrodov’, *Československá gynaekologie: časopis gynekologické a porodnické společnosti,* 47, 8 (1982), 635–635; Bedřich Srp *et al*., ‘Naše současná koncepce vedení předčasných porodů: výběr z referátů přednesených na sjezdu Čs. společnosti gynekologicko-porodnické, Košice, 20.-22.9.1984’, *Československá gynaekologie: časopis gynekologické a porodnické společnosti,* 50, 8 (1985), 585–6.

102 ‘II Symposium of Perinatal Medicine of the Socialist Countries: Streszczenia: Poznań, November 19th–21st., 1981 = abstracts’ (S.l: s.n., 1981).

103

Iván Birtalan and Attila Molnár , ‘Kissúlyú magzatot szült nők néhány életmódbeli sajátosságának vizsgálata.’ Magyar Nőorvosok Lapja, 48, 5 (1985), 285–9.

104

Karl-Eugen Ruckhäberle , ‘Die drohende Frühgeburt – Ursachen, Mechanismen und Strategie ihrer Prävention’, Zeitschrift für Ärztliche Fortbildung, 82 (1988), 15–18 3285602.

105 Karl-Eugen Ruckhäberle, ‘Prävention von Frühgeburten’, *Zentralblatt für Gynäkologie,* 5, 108 (1986), 265–9.

106 Ruckhäberle, *op. cit.* (note 104), 17.

107 Troszyński, *op. cit.* (note 1), 13–25.

108 For an overview, see Kateřina Lišková and Stanislav Holubec , ‘Women between the Public and Private Spheres’, in Wlodzimierz Borodziej et al. (eds), The Routledge History Handbook of Central and Eastern Europe in the Twentieth Century. Volume 1 (London: Routledge, 2020), 183–234.

109 Hofman, *op. cit.* (note 49).

110 Szenajch, *op. cit.* (note 80), 468.

111 Opitz, *op. cit.* (note 1), 23-24.

112 Experts’ suspicions arose because of East German’s abortion access, which was limited to medical conditions and criminal cases. Unlike the other three countries, which by the end of the 1950s had legalised abortion on social grounds, it was not until 1972 that East Germany adopted similar legislation, legalising abortion for women who requested it during the first twelve weeks of the pregnancy.

113 Brey and Wienold, *op. cit.* (note 1), 81.

114 Akkermann *et al*., *op. cit.*(note 1), 195–197.

115 Marcusson, *op. cit.* (note 45), 18.

116 Bielicka, *op. cit.* (note 53); Rozalia Osuch-Jaczewska and Jan Tomala , ‘Wpływ przebytych przerwań ciąży i kolejności ciąży na przebieg następnej ciąży i porodu oraz stan, dojrzałość, dystrofię i umieralność noworodków’, Ginekologia Polska, 50, 2 (1979), 127–33422060; Argay, Nemecskay, and Szombathy, ‘Borsod megye 1964. évi össz-koraszülésének elemzése, majd 4 év múlva a koraszülöttek után vizsgálata’; Iván Birtalan and Attila Molnár, ‘Kissúlyú magzatot szült nők néhány életmódbeli sajátosságának vizsgálata’, *Magyar Nőorvosok Lapja,* 48, 5 (1985), 285–89. Cited in footnotes before.

117 Holásek, *op. cit.* (note 64), 53–54.

118 Komorowska and Królikowska, *op. cit.* (note 66).

119

Milek Alterman and Janusz Kubicki , ‘Przebieg ciąży i porodu u kobiet stanu wolnego’, Ginekologia Polska, 38, 2 (1967), 133.6045009

120

Józef Kuczyński et al., ‘Kliniczna analiza 1000 porodów przedwczesnych’, Ginekologia Polska, 41, 12 (1970), 1317 5498368.

121 San. “A csecsemőhalálozás alakulása a fővárosban.” *Egészségügyi Dolgozó*, 5, 8 (1961), 3.

122

Antal Bőhm , ‘A többszörösen hátrányos helyzetű rétegek vizsgálata’, *Szociológia*, 3–4 (1981), 279–332 , here: 309–10.

123

László Velkey , ‘Psychés tényezők szerepe a születési súly alakulásában’, Orvosi Hetilap, 107, 12 (1966), 544–75930152; Velkey, *op. cit.*, (note 83).

124 Argay, Nemecskay and Szombathy, ‘Borsod megye 1964. évi össz-koraszülésének elemzése, majd 4 év múlva a koraszülöttek után vizsgálata’. Cited in footnotes before.

125

Vojtech Kliment et al., ‘Psychosociálne aspekty predčasných pôrodov’, Československá gynaekologie: časopis gynekologické a porodnické společnosti, 39, 3 (1974), 197 4452040.

126

H. Beránková , ‘Dvě příčiny předčasných porodů: výběr z referátů přednesených na sjezdu Čs. společnosti gynekologicko-porodnické, Košice, 20.-22.9.1984’, Československá gynaekologie: časopis Gynekologické a porodnické společnosti, 50, 8 (1985), 583 .

127 Beránková, *op. cit.*, (note 126), 583.

128 A. Janosikova, ‘Sociologické aspekty predčasných pôrodov’, *Československá gynaekologie: časopis Gynekologické a porodnické společnosti,* 48, 8 (1983), 612. On the correlation between a good family environment and prematurity, see Miroslav Vichnar , ‘Příspěvek k dlouhodobému sledování těžce nezralých dětí z hlediska dětské psychiatrie’, Československá pediatrie, 35, 2 (1980), 90 7371105.

129

Libuše Avratová, J. Martincik and Vít Unzeitig , ‘Perinatální úmrtnost z nezralosti plodu’, Československá gynaekologie: časopis Gynekologické a porodnické společnosti, 47, 1 (1982), 33 ; Jaroslav Antoš et al., ‘Výsledky rozboru příčin nedonošenosti na území OÚNZ Šumperk a závěry z hlediska morbidity matek’, Československá gynaekologie: časopis Gynekologické a porodnické společnosti, 41, 1 (1976), 23 1260883.

130

Jan Grzesiak , Tadeusz Pisarski and Andrzej Woźnica , ‘Społeczno-medyczne aspekty wcześniactwa. V. Wpływ niektórych sytuacji rodzinnych na częstość występowania porodów przedwczesnych’, Ginekologia Polska, 54, 2 (1983), 113–146873724.

