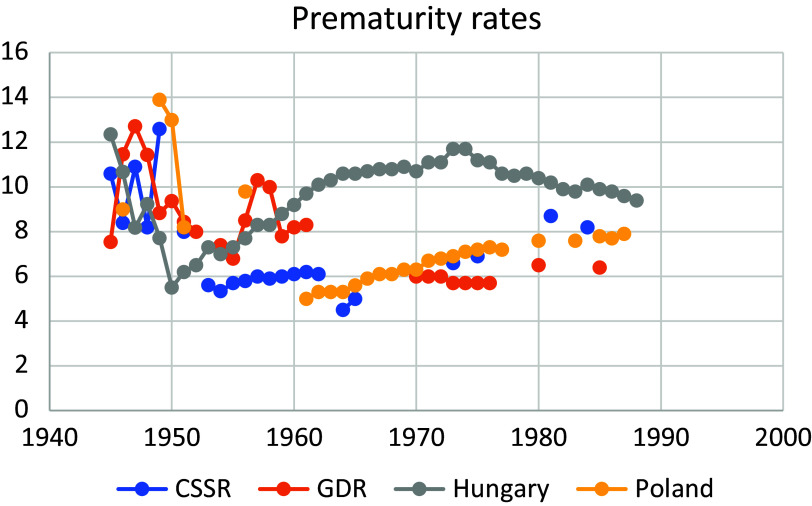# Work, marriage and premature birth: the sociomedicalisation of pregnancy in state socialist East-Central Europe – ERRATUM

**DOI:** 10.1017/mdh.2024.1

**Published:** 2024-01

**Authors:** Kateřina Lišková, Natalia Jarska, Annina Gagyiova, José Luis Aguilar López-Barajas, Šárka Caitlín Rábová

The Publisher apologises for the omission of Figure from the published paper. The Figure is given below.

**Figure 1:**